# Nanogenerator-Based Self-Powered Sensors for Wearable and Implantable Electronics

**DOI:** 10.34133/2020/8710686

**Published:** 2020-03-10

**Authors:** Zhe Li, Qiang Zheng, Zhong Lin Wang, Zhou Li

**Affiliations:** ^1^CAS Center for Excellence in Nanoscience, Beijing Key Laboratory of Micro-Nano Energy and Sensor, Beijing Institute of Nanoenergy and Nanosystems, Chinese Academy of Sciences, Beijing 100083, China; ^2^School of Nanoscience and Technology, University of Chinese Academy of Sciences, Beijing 100049, China; ^3^Center on Nanoenergy Research, School of Physical Science and Technology, Guangxi University, Nanning 530004, China; ^4^School of Material Science and Engineering Georgia Institute of Technology Atlanta, GA 30332-0245, USA

## Abstract

Wearable and implantable electronics (WIEs) are more and more important and attractive to the public, and they have had positive influences on all aspects of our lives. As a bridge between wearable electronics and their surrounding environment and users, sensors are core components of WIEs and determine the implementation of their many functions. Although the existing sensor technology has evolved to a very advanced level with the rapid progress of advanced materials and nanotechnology, most of them still need external power supply, like batteries, which could cause problems that are difficult to track, recycle, and miniaturize, as well as possible environmental pollution and health hazards. In the past decades, based upon piezoelectric, pyroelectric, and triboelectric effect, various kinds of nanogenerators (NGs) were proposed which are capable of responding to a variety of mechanical movements, such as breeze, body drive, muscle stretch, sound/ultrasound, noise, mechanical vibration, and blood flow, and they had been widely used as self-powered sensors and micro-nanoenergy and blue energy harvesters. This review focuses on the applications of self-powered generators as implantable and wearable sensors in health monitoring, biosensor, human-computer interaction, and other fields. The existing problems and future prospects are also discussed.

## 1. Introduction

In the past decades, wearable and implantable electronics (WIEs) have experienced a period of rapid development and are more and more important and attractive to the public [1–6]. Nowadays, wearable and implantable electronics have penetrated into every aspect of our lives, making people's lifestyles more efficient and convenient [7–10]. As the core components of WIEs, practical mobile sensors require integration ability with accessories and fabrics such as bracelets, watches, eyeglasses, necklace, or the implantation into the human body [11, 12]. However, their developments still need to overcome lots of challenges, such as how to reduce weight and size. Furthermore, for implantable electronics especially, flexibility is particularly necessary [13].

Another great limitation is the power supply. For full functions, most current practical mobile electronics still require outer power supplies usually batteries to provide power. The battery occupies most of the volume and weight, and periodic replacement of it will lead to electronic waste, physical burden, and financial strain to patients. Therefore, self-powered systems are imperative to practical wearable and implantable electronics [14]. Our bodies contain a variety of energy, including chemical, thermal, and mechanical energy, among which mechanical energy is the most abundant. For the purpose of utilizing this mechanical energy, a nanogenerator (NG) which can transform mechanical energy into electric energy was invented by Professor Zhong Lin Wang in 2006 and has made a remarkable progress in recent years [15, 16]. Typically, NGs can be divided into three types based on electricity generation mode, namely, piezoelectric nanogenerator (PENG), triboelectric nanogenerator (TENG) [17], and pyroelectric nanogenerator (PYENG) [18]. They have been widely used as micro-nanoenergy or blue energy harvesters and self-powered sensors [19–25]. Considering that more and more implantable and wearable electronic sensors have been employed by humans, developing NG-based technology is extremely attractive [26, 27].

For conformal integration with the skin or organs, extremely lightweight and flexible energy-collecting devices were of the essence. Piezoelectric materials have been chosen for preparing PENG, such as ZnO [28, 29], BaTiO_3_ [30], NaKNbO_3_, Pb(Zr_x_Ti_1-x_)O_3_ (PZT) [31], and polyvinylidene fluoride (PVDF) [32]. The structure of PENG has developed from single nanowires to films in order to obtain good stability and high output. The TENG has a wide selection of materials, and new designs of the structure are being constantly invented for higher and more stable electric performance [33, 34]. PENGs and PYENGs are the devices usually fixed on flexible support films such as polyimide (PI) and polyethylene terephthalate (PET) and encapsulated in biocompatible materials such as polydimethylsiloxane (PDMS) and polytetrafluoroethylene (PTFE) to achieve flexible and biosafety electronics [35].

In this review, we first gave an introduction of the NG principles. Then, the specific materials and devices of NGs were summarized. In addition, we focused on the applications of NGs as implantable and wearable self-powered sensors in health monitoring, biosensor, human-computer interaction, and other fields. Finally, the existing problems and future prospects were also discussed.

## 2. Mechanism of NGs

PENGs, TENGs, and PYENGs are three kinds of NGs based on different electricity generation mechanisms. The origin of NGs was based on Maxwell's displacement current theory [36]. In simple terms, a time-varying electric field leads to changed electric flux; then, displacement current is generated.

### 2.1. PENG

**Figure 1:**
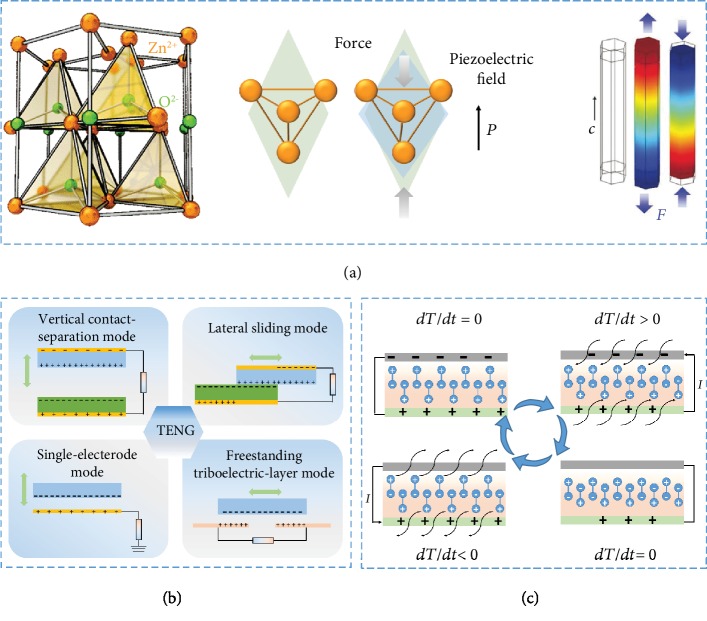
(a) Structure and working mechanism of the PENG based on ZnO nanowire. (b) Four fundamental working modes of TENGs. (c) Working mechanism of the PYENG.

Piezoelectric effect is an effect of generating internal potential (Figure 1(a)) [37]. When external force is applied, the mutual displacement of anions and cations in the crystal produces electric dipole moment, which can generate electric voltage difference in the tension direction of the material. The mutual transformation between mechanical force and voltage can be realized by this effect. PENG is an energy conversion device that utilizes the piezoelectric effect of nanomaterials. The existing PENGs are mainly composed of external loads, piezoelectric materials that can generate voltage potential and flexible substrates. PENGs can be divided into n-type material NGs and p-type material NGs. Taking zinc oxide (ZnO) nanowire as an example [38, 39], ZnO has wurtzite structure, in which Zn^2+^ and O^2-^ form tetrahedral coordination. One side of the nanowire is settled, and the other side is connected to a fixed electrode. In the beginning, the centers of the cations and anions overlap with each other. When the free end of the zinc oxide wire is subjected to deformation by the driving electrode, one side of the nanowire is compressed and the other side is stretched, resulting in piezoelectric potential. The Schottky barrier at both ends of the ZnO nanowires can store electrical energy inside the ZnO nanowires temporarily. Once the nanowire is connected to an external circuit, the electrons would be driven through the external load to achieve the new balanced state by the piezoelectric potential. Thereby, a continuous pulse of current could generate in the external circuit as the external force on the zinc oxide changes. The piezoelectric mechanism of another material is similar to ZnO.

### 2.2. TENG

TENG is based on the effects of friction electrification and electrostatic induction [40]. The basic principle is that two kinds of materials could carry the same amount of different charges after contact and separation due to different electron capture properties. When a circuit is connected to the peripheries of these two materials, a current can be generated due to the induced potential between the peripheries. According to the “contact” modes of the two friction materials and the linkage of external circuits, the working modes of TENGs were classified into four categories (Figure 1(b)) [41].

#### 2.2.1. Vertical Contact-Separation Mode

The two contact layers are charged with equal amount of opposite charges after contact. In the process of separation, the capacitance of the two contact layers with air as the medium decreases with the increase of space. At the same time, even if the negative contact layer is not conductive, the generated negative charges cannot move. As a consequence, in order to equilibrate the negative charges, the electrons in the metal electrode connected with the negative contact layer are transferred to the positive contact layer through the outer leading wire. On the contrary, as the capacitance increases, electrons near the positive contact layer flow back to the metal electrode on the backside of the negative contact layer.

#### 2.2.2. Lateral Sliding Mode

The overall model of the sliding NG is very similar to that of the contact-separation mode, except that one contact layer changes from contact and separation with the other layer to one pole sliding on the other layer. Because the change occurs as the two materials contact with each other, the charge from friction of the two materials would no longer be in alignment; that is, no capacitance between the two contacted materials would exist. However, with the change of sliding, the polarization region of the friction layer that has no contact after sliding will decrease and increase with the sliding overlap and separation of the two contact layers, which corresponds to the reciprocating movement of electrons used by the peripheral circuit to shield the electric field.

#### 2.2.3. Single-Electrode Mode

In the single-electrode theoretical model, a single electrode is connected to an external circuit at one end, which is different from contact split or horizontal slide. As shown in Figure 1(b), the charges generated from the two contact materials will charge the capacitor composed of one electrode and the other reference electrode. When the two contact layers are far away from or close to each other, the capacitance between the two contact layers will also change, which corresponds to the charges transferred between the two capacitive plates in the external circuit.

#### 2.2.4. Freestanding Triboelectric Layer Mode

Freestanding TENG is the optimization of single-electrode TENG. In order to utilize all the induced potential of the two electrodes on the single-electrode NG, an independent layer structure is designed that can put both electrode plates into the electrode structure. Theoretically, the upper limit of the efficiency can be restored to 100%. According to the contact mode of the contact layer, the independent layer NG can also be separated into contact-separation type and sliding friction type. And the mechanism of electric generation is similar to those of the vertical contact-separation mode and lateral sliding mode TENG.

### 2.3. PYENG

PYENG is an energy collection device that can convert heat energy into electrical energy by using nanomaterials with pyroelectric effects (Figure 1(c)) [42, 43]. Pyroelectric effect refers to a phenomenon that the spontaneous polarization of some crystals changes with the variation of temperature when they are heated, leading to the transformation of surface bound charge of the crystals. When the temperature does not change with time, the spontaneous polarization intensity of the crystal remains unchanged; no pyroelectric current is generated, as shown in Figure 1(c). However, once the temperature increases with time, the intensity of spontaneous polarization will decrease. As the crystal is connected to the external circuit, pyroelectric current will be generated in the circuit. When the temperature rises and finally reaches equilibrium, neither the temperature nor the spontaneous polarization of pyroelectric crystals will have continuous change, resulting in no pyroelectric current. If the temperature of the crystal changes, such as cooling the crystal, the spontaneous polarization of the crystal will increase, and the pyroelectric current will be generated in the external circuit until an equilibrium is reached. The continuous cycle could produce a constant current.

## 3. Materials and Devices

### 3.1. Piezoelectric Materials and Devices

Piezoelectric materials are functional materials with piezoelectric effect, which could generate electric current under external force or generate force and deformation under the action of current in turn. It is widely used in transducers to converse the mechanical energy into electrical energy. Since the piezoelectric effect was discovered by the Curie brothers in 1880, a variety of piezoelectric materials have been invented, which can be separated into the following two categories: (1) inorganic materials, such as ZnO [44], lead zirconium titanate (PZT) [45], barium titanate (BT), modified lead zirconate titanate [46], lead metaniobate, lead niobate barium lithium (PBLN), and modified lead titanate (PT) [47, 48]; (2) organic materials, such as polyvinyl chloride (PVC), polyvinylidene fluoride (PVDF) [49] and its derives [50], and some other materials [51–53]. All the materials have been studied in fabricating PENGs, as shown in Table 1. In the development of PENGs, the selection of materials is very important for the design and optimization of devices. ZnO and PVDF have low weight and great flexibility; they are suitable for large-scale deformation and multidimensional deformation applications, such as human body movement sensors. PZT, BaTiO_3_, and PMN-PT have high piezoelectric strain constant (d33) up to 3 times, 25 times, and 90 times of ZnO, respectively [39]. Therefore, PZT-, BaTiO_3_-, and PMN-PT-based devices are appropriate for energy harvesting.

#### 3.1.1. Inorganic Materials and Devices

Devices made of inorganic piezoelectric materials have been widely studied. Inorganic piezoelectric materials are divided into piezoelectric crystals and piezoelectric ceramics. Piezoelectric crystals generally refer to piezoelectric single crystals, which grow based on a long range of crystal space lattice. The crystal structure has no symmetrical center, leading to the piezoelectric properties. Piezoelectric ceramics generally refer to piezoelectric polycrystals, in which ferroelectric domains exist in the grains of ceramics. Under the condition of artificial polarization (reinforcing the direct current electric field), the spontaneous polarization is fully arranged in the direction of the external electric field and the residual polarization intensity is maintained after removing the external electric field, so it has macroscopic piezoelectric properties. The successful development of this kind of materials promoted the performance improvement of various piezoelectric devices, such as acoustic transducers and piezoelectric sensors.

**Figure 2:**
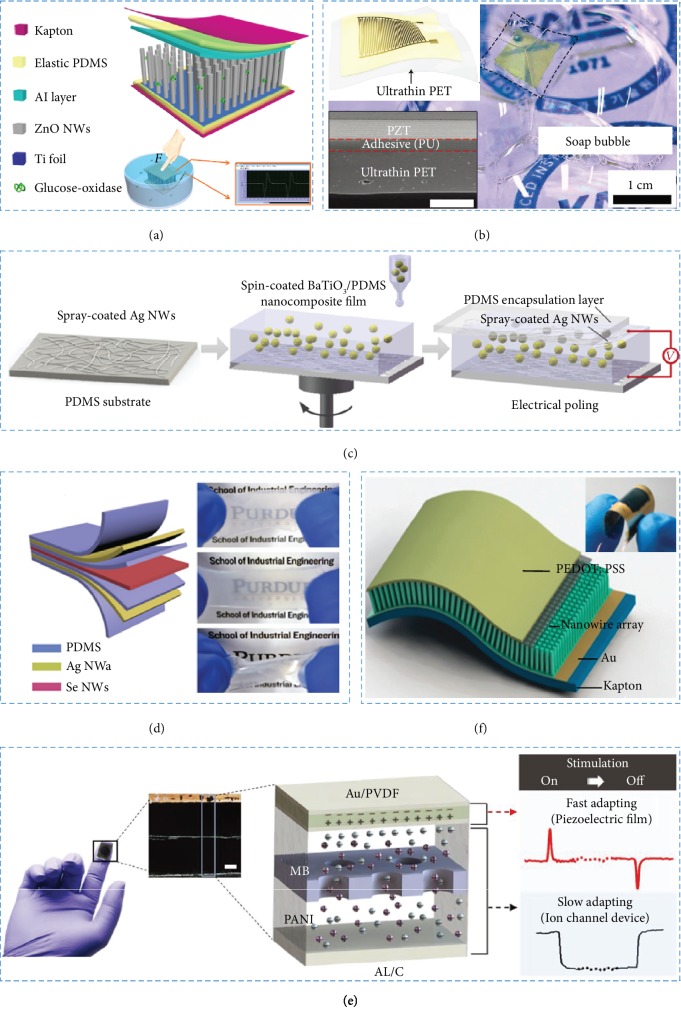
Piezoelectric materials were studied to fabricate NGs. (a) The e-skin based on ZnO nanowire could be driven by body motion. (b) The flexible pressure sensor is composed of ultrathin adhesive layer, flexible substrate, and PZT thin film. (c) The fabrication process of BaTiO_3_/PDMS-based PENG. (d) Se-PENG device could be stretched or twisted. NWs: nanowires. (e) The artificial cutaneous sensor is made from PVDF. MB: membrane; PANI: polyaniline; AL/C: aluminum foil coated with conductive carbon. (f) The flexible piezoelectric device is composed of PEDOT:PSS.

*(1) ZnO and Derivative*. Yang et al. report a flexible power generator with ZnO nanowire for the first time [54]. The wire was fixed in the flexible substrate, and both ends were closely adhered to the metal electrode. Basing on periodic releasing and stretching of the wire, under the strain of 0.05–0.1%, a repeated voltage output of up to 50 mV and the energy conversion efficiency of 6.8% could be produced. Furthermore, some piezo-biosensing unit matrixes of enzyme/ZnO nanoarrays have been employed in the biosensing process [55]. GOx@ZnO (GOx (glucose oxidase)) nanowire arrays have been applied in preparing a self-powered electronic skin (e-skin). Based on the piezo-enzymatic reaction coupling process of the nanowire, the voltage of e-skin is negatively correlated with the glucose concentration in solution. In addition, as shown in Figure 2(a), the output voltage of e-skin has a relationship with the concentration of lactate, uric acid, and urea in perspiration [56]. We believe that the research of these devices is likely to accelerate further development and application of self-powered systems.

*(2) PZT and Derivative*. Dagdeviren et al. fabricated a flexible PZT mechanical energy harvester (MEH) which could convert mechanical motion into energy in different positions of different animal models [45]. The sandwich-structured MEH was composed of a PZT film, a top electrode (Cr/Au), and a bottom electrode (Ti/Pt). The MEH could be utilized as a power supply for cardiac pacemakers in heart pacing with the energy conversion efficiency of ∼2%. By using PZT, Park et al. prepared a PENG-based pulse sensor [57]. The sensors contained three parts: one substrate of an ultrathin polyethylene terephthalate (PET), one piezoelectric layer of a PZT thin film, and an Au-integrated electrode formed on the PZT film (Figure 2(b)). The flexible device was adhered to the skin and could sense tiny pulse changes of the epidermis. The sensor has good mechanical stability, response time of 60 ms, and a sensitivity of 0.018 kPa^−1^. This is the earliest study about a self-powered piezoelectric pulse sensor. The same year, Kim et al. applied good-performance single-crystalline (1−x)Pb(Mg_1/3_Nb_2/3_)O_3_−(x)Pb(Zr,Ti)O_3_ (PMN-PZT) in driving a wireless communication of the healthcare system [58]. The PMN-PZT energy harvester could generate *in vivo* short circuit current and open circuit voltage about 1.74 *μ*A and 17.8 V, respectively, which were 17.5 and 4.45 times of the previous device fabricated by Dagdeviren et al. [45]. The energy harvester provided a new device for the diagnosis and treatment of biomedical applications.

*(3) Others*. As shown in Figure 2(c), an innovative device was prepared with silver nanowires, BaTiO_3_, and polydimethylsiloxane [59]. The devices exhibited great transparency and flexibility. They could be stretched, folded, or twisted. The NG showed a perfect linearity of input−output under the vertical stress-strain and the sensing property of detecting lateral tensile deformation up to 60%. Moreover, the as-fabricated device could collect touch energy from the body in great efficiency. The centimeter-scale PENGs were prepared to harvest energy from human motions by using molybdenum disulfide (MoS_2_) [60]. The PENGs were composed of MoS_2_ nanosheets, vertically grown hollow MoS_2_ nanoflakes (v-MoS_2_ NFs), and flexible substrate. Wu et al. discovered a solution-synthesized selenium (Se) nanowire with piezoelectric property which was sensitive to strain [61]. The length of the nanowires ranges from tens of *μ*m to 100 *μ*m, while the diameter is ~500 nm. Se-PENG device consists of multiple layer stacking of PDMS, Ag nanowire electrodes, and piezoelectric Se nanowires, which are highly flexible and stretchable (Figure 2(d)). Tellurium (Te) nanowires have also been studied as a kind of piezoelectric materials [62]. The device consisted of multiple layer stacking of ITO/PET, Te nanowires, and PDMS which were prepared. The Te-based piezoelectric device shows the excellent deformability which can withstand large degrees of mechanical deformation without fracture or cleavage. The flexible and ultrathin devices could be closely adhered to the human body, which is essential for cardiovascular or radial artery pulse monitoring. Therefore, the solution-synthesized Se nanowire and Te nanowire can be applied as new types of piezoelectric nanomaterial for self-powered biomedical sensors.

#### 3.1.2. Organic Materials and Devices

Organic piezoelectric materials are also known as piezoelectric polymers, such as PVDF (thin film) and other polar polymer piezoelectric (thin film) materials. These materials have great flexibility, low density, low impedance, and great piezoelectric voltage constant which promote the rapid development in hydroacoustic ultrasonic measurement, pressure sensing, ignition and detonation, and other applications. Though the organic materials have great piezoelectric voltage constant (g), they have low piezoelectric strain constant (d). Therefore, the organic material-based devices are not very suitable for working as active emission transducers.

*(1) PVDF*. PVDF is one kind of organic piezoelectric material and could also be used for fabricating PENG. The prepared device was composed by some slits in PVDF film and a polydimethylsiloxane (PDMS) bump, which could respond to multidirectional forces and convert strain energy [63]. As directions of the induced strain generating in PVDF varied with the changed direction of imposed force, the output generated became different. Yu et al. fabricated a shoepad NG based on PVDF nanofibers, which could harvest energy from walking or running [49]. The nanofabric mats were prepared to sandwich structure for better energy output of about 6.45 *μ*W when resistance of load was 5.5 M*Ω*.

In addition, a self-powered mechanoreceptor sensor composed of a piezoelectric film (Au/polyvinylidene fluoride (PVDF)), an electrode (AL/C), an electrolyte (polyaniline (PANI) solution), and a pore membrane (MB). The structure has an artificial ion channel system that mimics functions of slow adaption (SA) and fast adaption (FA) and can detect stimulation with broad frequency band and high sensitivity (Figure 2(e)). Discriminating signals are obtained for various types of touches and pressures with high sensitivity (0.21 V kPa^−1^ at static pressure) [64].

As the PVDF is transparent and flexible, PVDF-based PENG could also be applied in environment measurement. Fu et al. fabricated a breath analyzer for detection of internal diseases based on polyaniline/polyvinylidene fluoride (PANI/PVDF) piezo-gas-sensing arrays [65]. By combing the PVDF with in-pipe gas flow-induced piezoelectric effect and PANI electrodes with gas-sensing properties, the devices can convert exhaled breath energy into electric output. A sensor based on a single-electrode PVDF PENG (SPENG) has been employed. Due to the use of cost-efficient PVDF and electrospinning, the preparation method is extremely simple. Therefore, this method can be manufactured on a large scale and at a low cost. The steady-state pressure and temperature-sensing performance of electronic skin were simultaneously measured, which ensured that the SPENG could be used for truly autonomous robots [66].

*(2) P(VDF-TrFE)*. A self-powered sensor with P(VDF-TrFE) nanowires located in the nanopores of an anodized aluminum oxide (AAO) template is prepared in Figure 2(f) [67]. When the device was bent, the device could have a current density of 0.11 mA·cm^−2^ and a max voltage of 4.8 V. The devices were highly sensitive, which enabled that the devices could be applied for monitoring vital signs, for example, breath, heartbeats, and pulse and finger movements. The P(VDF-TrFE) nanowires could be employed for forming a cellular fluorocarbon piezoelectric pressure sensor (FPS) with ultrahigh sensitivity as well [68]. The flexible FPS has a rapid response time of 50 ms, a great sensitivity about 7380 pC·N^−1^ in the subtle-pressure regime (<1 kPa), a high stability up to 30,000 cycles, and an extremely low limit of detection about 5 Pa.

Furthermore, combining with multiwalled carbon nanotube, a piezoelectric film with high sensitivity, excellent mechanical strength, and good piezoelectricity has been synthesized [69]. The composite film had a sensitivity approximately 540 mV·N^−1^, a Young's modulus of 0.986 GPa, and a piezoelectric coefficient about 50 pm·V^−1^, which could be made into a piezoelectric pressure sensor to detect tiny physiological information.

### 3.2. Triboelectric Materials

**Figure 3:**
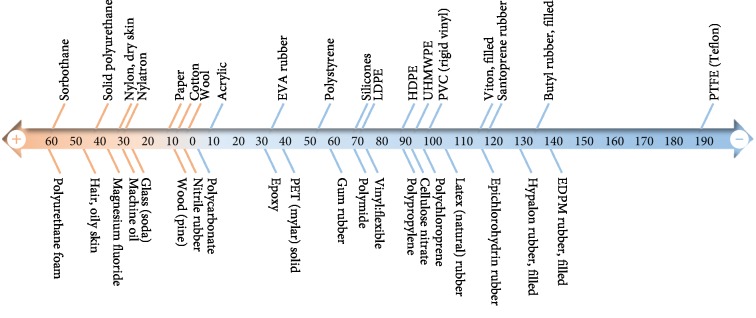
The triboelectric series of traditional materials. The materials that go in the “positive” direction are more inclined to lose electrons and in the “negative” direction are easier to gain electrons.

Almost all the materials have triboelectric effect, which include silk, polymer, metal, and wood. All these materials can be used as friction layers, resulting in a wide selection range of materials for TENGs. The materials usually have different frictional electric sequences (Figure 3). When the difference between two materials is bigger, the corresponding charge transfer amount is larger, and the output of the prepared TENG is higher. The triboelectric materials could be divided into four kinds: polymer-metal [72–80], polymer-polymer [81–84], polymer-semiconductor [85–87], and others [88–92]. The summarization of materials and fabrication of TENGs are shown in Table 2.

#### 3.2.1. Polymer-Metal

**Figure 4:**
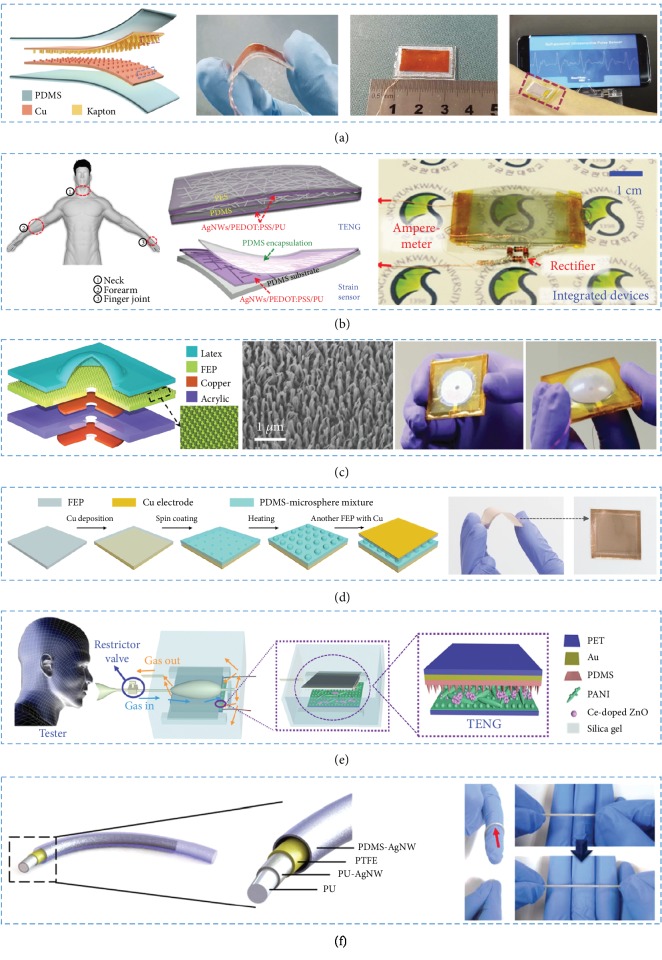
The triboelectric materials and devices. (a) The bendable SUPS is made from Cu-Kapton and could be placed over the radial artery. (b) The patchable integrated devices are made from PDMS-AgNWs/PEDOT:PSS/PU and tied on the neck, forearm, and finger joint. (c) The M-TES consists of FEP nanorod arrays. (d) The pressure sensor has a size of 33 × 33 mm^2^ and was fabricated by FEP-PDMS. (e) The human respiration-driven system was prepared by PDMS-PANI. (f) The TENG device based on PU-AgNW has a coaxial linear structure and could be stretched to strain of ~50%.

The energy generated by human walking and running was collected by a multilayered attached electrode TENG [93]. A thin aluminum foil was used as both electrode and friction layer, the fluorinated ethylene propylene (FEP) film was one friction layer and the copper deposited on it served as the second electrode. The TENG in shoe insoles could be driven to generate voltage of about 700 V output and short circuit transferred charge of 2.2 mC. It provided a new kind of TENG which could work in different conditions. Ouyang et al. fabricated a self-powered pulse sensor (SUPS) based on a triboelectric active sensor [94]. The SUPS was composed of four parts: friction layers, electrodes, spacer, and encapsulation layer (Figure 4(a)). Nanostructured Kapton (n-Kapton) film served as one triboelectric layer, and the Cu layer deposited on its backside acted as one electrode. The nanostructured Cu (n-Cu) film served as both triboelectric layer and electrode. The as-fabricated SUPS was ultrasensitive and low cost, which is important for sensors.

Furthermore, as shown in Figure 4(b), Hwang et al. proposed a self-powered strain sensor using a conductive elastomer of poly(3,4-ethylenedioxythiophene):polystyrene sulfonate (PEDOT:PSS)/polyurethane (PU) with low-density AgNW multifunctional nanocomposite materials [95]. The fabricated sensor had great flexibility, high stretchability, and excellent strain-responsive electrical properties.

Afterward, as the requirement for implantable devices, Li et al. fabricated biodegradable (BD) implantable TENGs (iTENGs) [96]. The Biodegradable Polymer 1 (BDP1) layer acted as one friction layer, and the Au evaporated at hemisphere array is the other triboelectric layer. The *in vivo* and *in vitro* output voltage of BD iTENGs were 2 V and 28 V, respectively. The researchers provided a medical device with controllable degradation property.

#### 3.2.2. Polymer-Polymer

A self-powered membrane-based triboelectric sensor (M-TES) was prepared to measure the variation of pressure [97]. Acrylic sheet acted as the substrate, and the copper deposited on both sides of it was applied as the top electrode and back electrode (Figure 4(c)). The friction layers were FEP membranes attached onto the top electrode and a latex film on the top of the sensor. According to the air-conducting channel at the center of the device, the sensor could harvest signals. Furthermore, Liu et al. fabricated TENGs which had hemispherical microstructures [98]. The TENG was low cost, highly sensitive, and easy to fabricate, which enabled it to be applied as a self-powered pressure sensor. Both electrodes were Cu films, and the triboelectric layers were FEP and PDMS microsphere mixture (Figure 4(d)). The sensors could measure pressure with the continuous process of contact-separation between expandable microsphere array and FEP film.

#### 3.2.3. Polymer-Semiconductive Materials

Qiu et al. reported a flexible and soft TENG, which could be applied for wearable smart healthcare [99]. The TENG had polymerized polyaniline (PANI) as electrodes and PA6 and PVDF as triboelectric layers. The polycaprolactone (PCL) was introduced to add the adhesion between friction material and electrode layer. The TENG could produce a short circuit current of 200 *μ*A and an open circuit voltage of 1000 V under a frequency of 2.5 Hz, which could continuously drive about 1000 LEDs.

Wearable TENGs with both multifunctionality and comfortability have become an attractive research for portable electronic devices in recent years. A TENG that consisted of two nanofiber membranes with arch structure was introduced [100]. One membrane thermoplastic polyurethane (TPU) was supported by Ag elastic fabric PVDF, and the other was supported by conducting fabric. The as-fabricated TENG could harvest energy from various types of irregular movements with the stretch ability from the TPU/Ag layer. The stretchable and tailorable TENG has been applied in both biomechanical monitoring and energy harvesting. Furthermore, Wang et al. also fabricated a TENG for NH_3_ detection [101]. A flexible and modified PDMS film was applied as triboelectric layer, and the Au deposited on the backside of PDMS was selected as one electrode of TENG (Figure 4(e)). Ce-doped ZnO-PANI nanocomposite film was used as NH_3_-sensing film, the other electrode and triboelectric layer. Due to the contact-separation of the PDMS layer and Ce-doped ZnO-PANI film, the TENG could have wide NH_3_ detection ranges and high sensitivity, which is important for identifying different breathing frequencies and patterns. The sensor provided a self-powered respiratory monitoring device for the detection of exhaled NH_3_.

Basing on some other semiconductive materials, Xia et al. fabricated a self-powered temperature sensor based on TENG containing triboelectric layers of PTFE and PVDF. The copper foil was selected as the conductive electrode [85]. The voltage output of the sensor was positively correlated with temperature. Therefore, the sensor could be applied in temperature monitoring.

#### 3.2.4. Others

In Guan et al.'s work, a self-healing triboelectric nanogenerator (HS-TENG) was fabricated with poly(vinyl alcohol)/agarose hydrogel, which was highly deformable and self-healable [90]. Multiwalled carbon nanotubes (MWCNTs) and active polydopamine particles were doped into the hydrogel resulting in self-healing of TENG in 1 min with near-infrared light (NIR) irradiation. A single-electrode HS-TENG could produce a *V*_oc_ of 95 V, an *I*_sc_ of 1.5 mA, and a *Q*_sc_ of 32 nC. The as-fabricated devices could be applied as wearable electronics. Shi et al. provided a body-integrated self-powered system (BISS) [102]. Basing on the electrode adhered to the skin, the BISS could harvest biomechanical energy from triboelectrification between soles and floor and the electrification of the human body. The *V*_oc_, *I*_sc_, and *Q*_sc_ varied with different states of movement. For example, the BISS could produce an output of 82 V, 0.5 *μ*A, and 19 nC on tiptoe and 134 V, 1.1 *μ*A, and 90 nC in stepping, respectively. The BISS supplied a fresh device for energy harvesting.

In addition, a series of coaxial fibers were applied for fiber nanogenerator (FNG) [103]. The Ag nanowire (AgNW) and polyurethane (PU) fiber with PTFE coating were selected as core parts, the polydimethylsiloxane-AgNW (PDMS-AgNW) film was used as the sheath electrode, and a cavity was introduced to provide contact-separation for core fiber and sheath (Figure 4(f)). The as-prepared FNG was so flexible and stretchable that it could be adhered to any surface to make some senses.

### 3.3. Pyroelectric Materials

At present, pyroelectric materials can be divided into three types: (1) monocrystalline materials: triglyceride sulfate (TGS), deuterium triglyceride sulfate (DTGS), potassium tantalum niobate (KTN), etc.; (2) polymer organic polymer and composite materials: PVDF, lead titanate compound (PVDF-PT), polyvinylidene fluoride with lead zirconate titanate compound (PVDF-PZT), etc.; and (3) metal oxide ceramics and thin film materials: ZnO, BaTiO_3_, lead magnesium niobate (PMN), barium strontium titanate (BST), etc. Based on pyroelectric materials, PYENGs were prepared which are more sensitive to the variation of temperature than PENGs and TENGs. Therefore, PYENG can be directly applied as a medical information sensor. Moreover, it can also harvest energy derived from the temperature difference between the body and ambient temperature and then provide electricity for other medical information sensors.

### 3.4. Materials in Hybrid Nanogenerators

Hybrid NGs were composed of at least two kinds of NGs to obtain better performance [110–113]. The summarization of wearable and implantable Hybrid NGs is shown in Table 3. A hybrid generator (HG) composed of a TENG, an EMG, and a PENG was provided for energy harvesting from human walking [114]. The PTFE and nylon were selected as the triboelectric layers of TENG and could supply energy basing on triboelectric effect and electrostatic induction. A sponge was placed between a planar coil and an NdFeB magnet to separate the coil from the magnet. As the device was pressed by the foot, the coil was close to the magnet and provided energy from magnetic induction. The PENG was based on ZnO nanowires, which has been widely used in fabrication of PENG. By integrating three generators, the efficiency of energy storage was highly increased.

**Figure 5:**
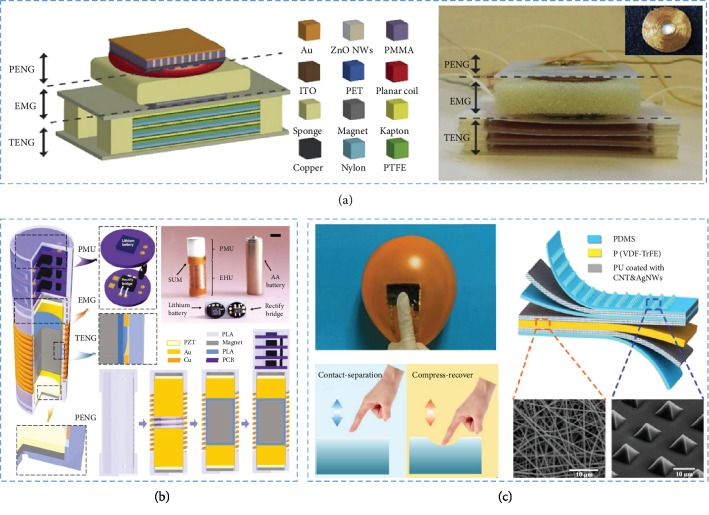
Hybrid NGs and their materials. (a) The hybrid generator is made from PENG, EMG, and TENG. (b) The SUM contains PMU, PENG, and TENG which were assembled into a dotted box. (c) The hybrid NG is composed of TENG and PENG.

As shown in Figure 5(b), self-charge universal modules (SUMs) were prepared with all of TENG, EMG, PENG, and energy management component gathered in a polylactic acid (PLA) tube [115]. Copper coils were twined around the tube, and an NdFeB permanent magnet (*d* = 10 mm, *h* = 15 mm) was positioned in the middle chamber of the PLA tube with magnetic levitation from another two reverse small magnets (*d* = 10 mm, *h* = 1 mm) fixed on both ends of the tube. Two Pb(Zr_1−x_Ti_x_)O_3_ (PZT) ceramic sheets (*d* = 10 mm, *h* = 0.5 mm) were settled in beyond of the two top magnets. With the mobilizable magnet striking the top magnets, the PENG could produce output based on piezoelectric effect. The nanostructured PTFE film on the surface of the mobilizable magnet was applied as the sliding friction layer. Two separated Kapton thin films that adhered to the inner wall of the PLA tube served as another friction layers. Two Al thin sheets seated beyond both PZT films were applied as electrodes. Under a shaking frequency of 5 Hz, the SUM can harvest an output power of 2 mW·g^−1^. Furthermore, the SUM could work for a long time under continuously jumping or running.

A flexible hybrid device basing on a single-electrode TENG and a PENG had been fabricated as shown in Figure 5(c) [116]. A PDMS film was introduced to isolate the PENG and TENG layers for ensuring the high output of the TENG. The composites of P(VDF-TrFE) and PU served as the piezoelectric membrane and electrodes, respectively. The TENG and PENG parts could produce power densities of 84 *μ*W·cm^−2^ and 0.11 *μ*W·cm^−2^ under compression, respectively. Because the piezoelectric materials had high sensitivity, the device could be suitable in any position of the body for real-time monitoring of the human physiology, which is vitally important for healthcare monitoring systems and self-powered e-skins.

The island-structured electromagnetic shielding hybrid nanogenerator (ES-HNG) was composed of a single-electrode mode TENG and several PENGs [117]. A layer of conductive antielectromagnetic radiation fabric (AEMF) among two layers of rubber was selected to prepare TENG. Woven with Ag-coated fabric fiber, the AEMF could be applied as wearable electronics. The as-fabricated ES-HNG could be used not only as an energy harvester but also as a health monitor.

A hybridized NG (HG) for scavenging airflow energies was composed of TENG and EMG, where the TENG contains the friction layer of PTFE film and Kapton film and the Cu on the backside of both friction layers acted as the electrode [118]. The EMG had a planar coil and a magnet fixed on the Kapton film. The HG would work with Kapton film vibration. With the air flowing in a speed of about 18 m·s^−1^, the hybridized nanogenerator could generate the largest output powers of 3.5 mW for one TENG at a loading resistance of 3 M*Ω* and 1.8 mW for one EMG at a loading resistance of 2 k*Ω*, respectively.

### 3.5. Fabrication Strategy for NG-Based Sensors

The basic principle of these self-actuated sensors is to use the change in the relative position of the two friction layers or center of positive and negative ions caused by mechanical trigger to induce specific changes in the voltage and current output. Besides, temperature sensors are more easily realized by PYENG because the output of it is closely related to temperature change.

The first application of NG is acting as self-actuated wearable pressure/tactile sensors. Information about external forces (including vibration, motion, and pressure) can be acquired by analyzing NG's output signals. The TENG-based sensor is more sensitive at low pressure. The possible explanation is that at high pressure, the gap between the two friction layers of contact-separation TENG closes, resulting in inseparability, while a PENG-based sensor can function at relative high-pressure environment. A mechanical motion can be represented by a series of parameters, including distance, acceleration, velocity, and angle. A TENG with different structures can be used to extract energy from different forms of mechanical motion, such as linear sliding and turning. We find that open circuit voltage of NG can be used for static measurements of pressure applied, while short circuit current can be used for dynamic measurements of pressure loading frequency or acceleration. In order to realize self-driven touch imaging, we can integrate multiple NG sensor units into an array structure for multichannel measurement.

In addition, NG can also be utilized as a wearable environmental/chemical sensor to detect the concentration of certain chemicals, the intensity of ultraviolet light, humidity, temperature, and other factors. The surface charge density of the friction layer is also an important factor affecting the output performance of the TENG, which is affected by many environmental factors. The change of external environment such as light and humidity or chemical substances will alter the chemical potential of the friction surface so that the surface charge density generated when it contacts with another friction layer will change, which would affect the electrical output performance of the TENG. In this way, we can distinguish changes in the concentration of certain chemical substances, such as heavy metal ions, phenol, catechin acid, and ultraviolet intensity, by detecting the output signal of the generator. The sensor prepared by this method has high sensitivity and important application value in environmental and chemical detection.

The application of NG *in vivo* is more complex and challenging than *in vitro* because encapsulation is obligatory. Self-powered NG sensors can be implemented by analyzing the corresponding electrical signals generated by excitation of the *in vivo* organs or tissue. Due to its sensitivity to low-frequency excitation, NG becomes an effective way to detect vibration and biological signals related to human health. The amplitude, frequency, and period information of voltage and current signals generated by implantable TENG or PENG are directly related to the mechanical input behavior of devices. Specifically, the voltage signal is related to the amplitude of mechanical vibration and the current signal directly reflects the dynamic process of mechanical vibration. Therefore, TENG and PENG can be used in detecting sound waves, heartbeats, and cardiovascular detection.

## 4. Biomedical Applications

### 4.1. Physical Sensors for Wearable Electronics

Wearable electronics could be closely adhered to the body or woven together with clothing. Combining with hardware equipment, software support, and data interaction, the NGs could implement their various functions as physical sensors [120–123]. In recent years, NGs have become smart and wearable as power sources or various sensors on different positions of the body. According to specific functions, the wearable NGs can be classified into four types: (i) tactile sensor, (ii) motion sensor, (iii) strain sensor, and (iv) others.  Table 4 summarizes various wearable electronics, including the sensor types, function in the sensing system, output voltage and sensitivity, device size, monitoring position, and constituent materials.

#### 4.1.1. Tactile Sensors

**Figure 6:**
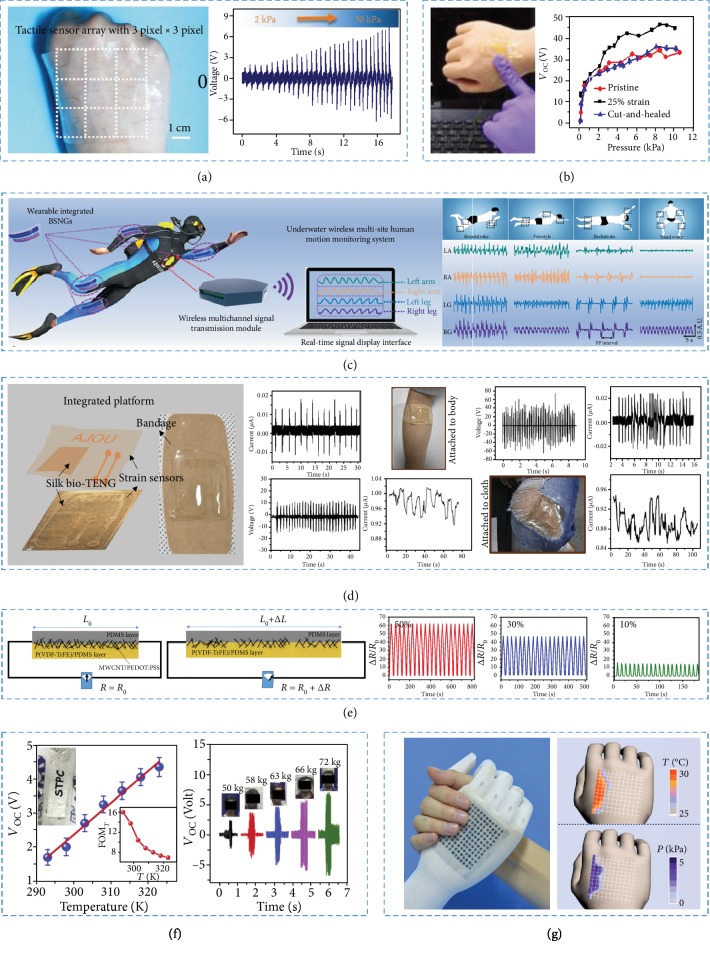
The NGs were applied as physical sensors for wearable electronics. (a) The voltage outputs of the 3 × 3 pixel tactile sensor varied with the pressure. (b) An EHTS-TENG-based tactile sensor was provided. (c) Underwater wireless multisite human motion monitoring system based on TENGs. (d) The integrated devices of the strain sensor and the bio-TENG were provided in measuring various human motions. (e) SMF fiber worked as a strain sensor to 20 cycles of loading and unloading from *ε* = 0% to 10%, 30%, and 50%. (f) The STPC was used to test different bending angles, temperatures, and weight. (g) MFSOTE devices were applied as flexible dual-parameter temperature–pressure sensors.

NGs could be applied as both power sources and self-powered tactile sensors [124–129]. Wen et al. reported a stretchable wrinkled and transparent TENG and applied it in tactile sensing [130]. The combined 3 × 3 pixel tactile sensor array was used in mapping the touch location and recording the shape of the object contacted with the sensor array (Figure 6(a)). As shown in Figure 6(b), a self-healed and flexible EHTS-TENG had been used in measuring different contact pressures with a great sensitivity [131]. The real-time signals generated in original state, 25% stretching, and after healing were recorded. As the contact pressure arose, the contact of two friction layers became more full and the thickness of friction layers decreased, resulting in an increase of the output voltages. The self-healing, flexible EHTS-TENG-based tactile-sensor had a great potential in human–device interfaces. A soft skin-like TENG (STENG) has been prepared as not only an energy harvester but also a tactile sensor with ionic hydrogel and hybridizing elastomer as the components [132]. The STENG could produce a peak power density of 35 mW·m^−2^ and drive wearable electronics with energy from mechanical motions. Moreover, the STENG was sensitive to pressure and could be employed in touch/pressure sensing.

#### 4.1.2. Motion Sensors

NGs could also be applied for motion monitoring [119, 133, 134]. Zou et al. reported a bionic stretchable and flexible NG with a structure of ion channels for underwater energy harvesting and human motion monitoring as shown in Figure 6(c) [135]. The bionic stretchable NG had been demonstrated in human body multiposition motion monitoring and undersea rescue systems.

Basing on the contact electrification and electrical induction from the body, Zhang et al. provided a new universal body motion sensor (UBS) to detect motions [136]. The contact electrification and electrical induction could produce a potential difference from variable charges between the body and the ground, which is closely connected to different body motions from the toes, feet, legs, waist, fingers, arms, and head. The as-fabricated devices have a great potential in healthcare, such as monitoring the conditions of people with Parkinson's disease (PD) or quantitatively supervising the recovery of those with a leg injury.

A flexible self-powered fabric-based multifunctional and waterproof TENG (WPF-MTENG) was introduced as both a waterproof wearable energy harvester from human motions and a motion sensor from finger touching or releasing [137]. By integrating into a music player system, a remotely controlling the system could be achieved with a series of WPF-MTENGs. The WPF-MTENGs were prepared into several buttons related to “Play/Stop,” “Last/Next,” and “Vol down/up” in a textile. As the button “Play” was pressed, the music was played; and the “Next” key could control the switching of songs. In addition, the WPF-MTENG could be made into smart bedspread to sense sleeper motions. The provided WPF-MTENG had great mechanical properties, outstanding stability, and excellent universality in different conditions, which prevail over the previous wearable devices. Therefore, the WPF-MTENG-based electronics could be provided for energy harvesting and motion detecting.

A PVDF-based single-electrode PENG (SPENG) was fabricated in steady-state sensing pressure and cold/heat with one integrated unit [138]. Therefore, a single unit could obtain the two signals simultaneously. The SPENG was superior to the previous e-skins based on a STENG, which could detect not only temperature variations but also dynamic movement at the same time. This might due to thermal-sensing signals appearing as pulse signals, while the obtained piezoelectric signals expressed as square wave signals. The fabricated sensor was flexible, transparent, and self-powered, which could be used for truly autonomous robots.

#### 4.1.3. Strain Sensors

Many NGs have played roles in strain sensors [106, 139–142]. A flexible and textile TENG for energy harvesting has proved the possibility as a strain sensor for strain sensing [109]. The integrated device was prepared in a single silk chip and could be adhered to the skin or fabrics to collect the biomechanical energy and detect strain at the same time (Figure 6(d)). The device was low cost, biocompatible, flexible, and transparent, enabling the usage as wearable bioelectronics, touch sensor, and seamless human-machine interface.

He et al. fabricated a height-varying multiarch strain sensor with wearable devices [143]. The strain sensing ranged from 10% to 160%. Furthermore, the arch-shaped strain sensors mounted on the human fingers can be applied in hand gesture monitoring for American Sign Language interpretation and robotic hand controlling. Additionally, several textile-based sensors located at different body parts can be used to track human activities.

Ryu et al. fabricated a stretchable strain sensor (Figure 6(e)) [144]. A strain of 10-50% in the top electrode had been measured to verify the strain-sensing properties which was suitable for the most wearable electronics. The resistance of the electrode increased when the stretch ratio enlarged. The electrode revealed a great sensing stability and excellent repeatability after 1000 cycles under a strain of 50%.

#### 4.1.4. Others

There are some other sensors based on NGs [108, 145]. As shown in Figure 6(f), a flexible self-charging triboelectric power cell (STPC) was prepared [146]. Under a cyclical pressure of 1 kPa, the STPC exhibited excellent output performances of 0.67 *μ*W (*P*_max_), 3.82 V (*V*_oc_), and 0.20 *μ*A (*I*_sc_), respectively. According to the results, the *V*_oc_ was highly sensitive and linearly responsive to the number of folding (*N*), externally applied weight (*W*), and temperature (*T*). As shown in Figure 6(f), *dV*_oc_/*dT* and *dV*_oc_/*dW* of STPC were found to be 0.093 V·K^−1^ and 0.2 V·kg^−1^ in temperature and weight range of 293–323 K and 50–72 kg, respectively. The STPC provided a highly sensitive sensor which could play a role in daily life.

A new kind of highly sensitive triboelectric sensors (“TESs”) with an interlocked hierarchical microridge structure was applied for detecting dynamic stimuli ranging from low to high frequency and high-frequency dynamic forces [107]. The TESs revealed a fast response, which could be applied in sound sensing. The TESs could receive the acoustic waveforms in different speeds and demonstrated time-dependent triboelectric output voltage variations. Except for identifying low sound pressure level (SPL) of human voice (<78 dB), the TESs can also be used in distinguishing different frequency domains of male and female voices with high reproducibility and reliability. Therefore, the devices will be applied in biometric security systems.

Microstructure-frame-supported organic thermoelectric (MFSOTE) devices were composed of microstructured frames and thermoelectric materials (Figure 6(g)). They have been applied as flexible dual-parameter temperature–pressure sensors. The self-powered sensors have great temperature-sensing accuracy less than 0.1 K and pressure sensitivity of more than 20 kPa^−1^. Therefore, the MFSOTE devices are promising as robotics and health-monitoring products [147]. Furthermore, PYENGs can be provided for scavenging energy of human respiration as self-powered human breathing and temperature sensors [148].

### 4.2. Implantable Biomechanical Sensors

**Figure 7:**
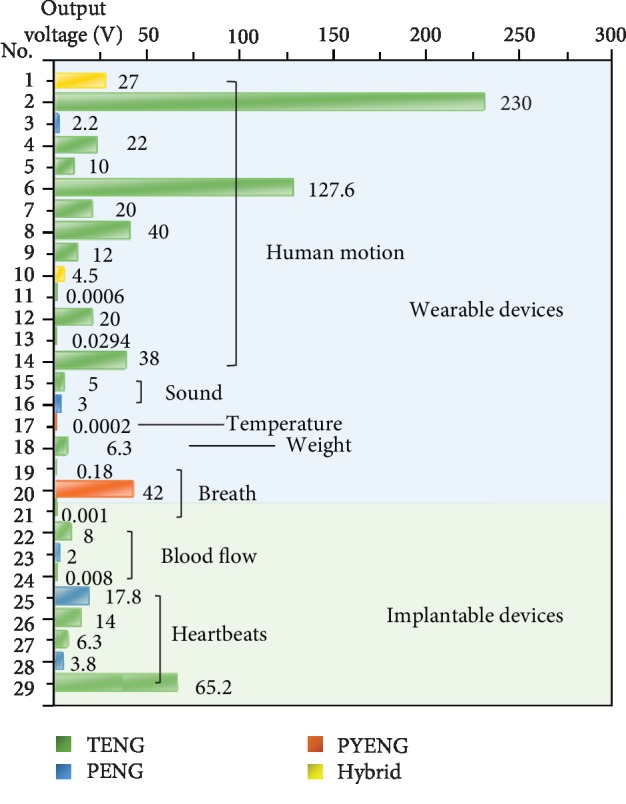
The output voltage of wearable and implantable NGs. Ref: No. 1: Ha et al. [107]; 2: Hou et al. [119]; 3: Kar et al. [128]; 4: Wen et al. [130]; 5: Zou et al. [135]; 6: Lai et al. [137]; 7: Yang et al. [106]; 8: Gogurla et al. [109]; 9: Wen et al. [130]; 10: Zhu et al. [134]; 11: Xu et al. [142]; 12: Lai et al. [131]; 13: Song et al. [139]; 14: Lan et al. [141]; 15: Ha et al. [107]; 16: Yan et al. [138]; 17: Zhang et al. [147]; 18: Karmakara et al. [146]; 19: Zhao et al. [129]; 20: Xue et al. [148]; 21: Li et al. [70]; 22: Ma et al. [105]; 23: Cheng et al. [71]; 24: Liu et al. [151]; 25: Kim et al. [58]; 26: Zheng et al. [104]; 27: Ma et al. [105]; 28: Dagdeviren et al. [45]; 29: Ouyang et al. [152]

Self-powered sensors are applied not only as wearable electronics but also as actively implantable biomechanical sensors (Figure 7). Since the motion energy of organs could be harvested by NGs, the outputs of NGs are strongly related with many biomedical signals, such as electrocardiogram (ECG), heart rate, blood pressure, velocity of blood flow, and respiratory rate and phase.  Table 5 summarizes various implantable biomechanical electronics for blood pressure sensing and cardiac and respiratory sensing. In addition, NGs also show great potential in the implantable electrical stimulation systems, such as the muscle, peripheral nervous system, and brain [149, 150].

#### 4.2.1. Blood Pressure Sensors

Hypertension is a chronic and persistent disease, which can greatly increase the incidence of heart failure and cerebrovascular diseases. According to statistics, about fifty percent of the global cerebrovascular disease and nearly half of the occurrence of ischemic heart disease are related to hypertension. The burden of hypertension is gradually increasing with the aging of the population. Implantable blood pressure (BP) monitors could not only provide early diagnosis and accurate assessment of diseases and drugs but also reduce medical costs. Through the study, researchers have found that the self-powered BP sensor which can avoid energy consumption has huge advantages. Such sensors could collect the pulse of the aorta and power the sensor directly. However, due to the limited space in the organism and the fragile aorta, the device should be ultrathin, flexible, and stable. In this regard, researchers have carried out a series of studies.

**Figure 8:**
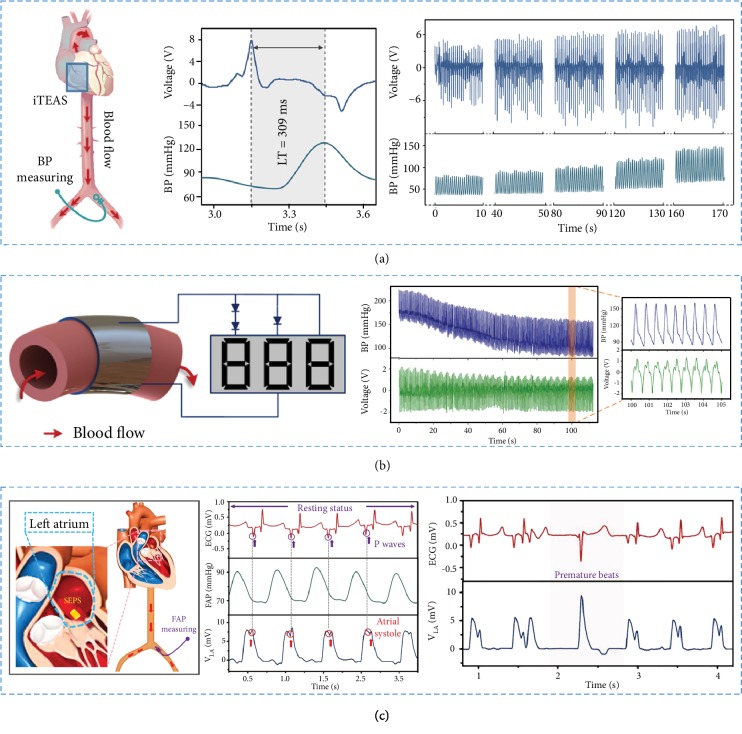
The implantable devices were applied in BP sensing. (a) The devices were applied for monitoring the velocity of blood flow. (b) The implantable, self-powered, and visualized BP monitoring system was fabricated. (c) SEPS was implanted into the left atrium, and a commercial arterial pressure sensor was placed in the right femoral artery to measure the ECG and the SEPS outputs.

An arterial pressure catheter was settled in the right femoral artery and connected to a multichannel data acquisition system with a transducer as shown in Figure 8(a) [105]. With the epinephrine injected, the peak output voltage of iTEAS increased accordingly with the variation of BP. In addition, the peak voltage decreased along with the BP going down. The devices showed an excellent correlation between the output voltage and the systolic blood pressure with a sensitivity of 17.8 mV·mmHg^−1^; the linearity is *R*^2^ = 0.78.

As shown in Figure 8(b), Cheng et al. fabricated a piezoelectric thin film- (PETF-) based BP monitor which was self-powered and implantable [71]. The studied stresses of the aorta wall and the electric potential from device were linearly correlated to systolic BP. The PETF was implanted into adult Yorkshire porcine, obtaining a sensitivity of 14.32 mV·mmHg^−1^ and an excellent linearity (*R*^2^¼ 0.971). With this device, the hypertension status could be warned visually in self-powered real-time monitoring. Therefore, as both energy harvester and biomedical sensor, the devices had a great potential in implantable healthcare monitoring.

On the basis of TENG, a self-powered, flexible, and miniaturized endocardial pressure sensor (SEPS) showed in Figure 8(c) was provided by Liu et al. [151]. By integrating the SEPS with a surgical catheter, a minimally invasive implantation could be achieved in the left ventricle and left atrium of a porcine model. The as-fabricated SEPS was highly sensitive (a sensitivity of 1.195 mV·mmHg^−1^) and mechanically stable and showed a good response to any pressures. In addition, the SEPS had been applied in detecting cardiac arrhythmias, such as ventricular premature contraction and ventricular fibrillation. The self-powered implantable device had great potential in monitoring and diagnosing cardiovascular diseases.

#### 4.2.2. Cardiac and Respiratory Sensors

Although the NG has been widely applied as blood sensors, it has great potential as a self-powered cardiac and respiratory sensor. They were implanted to directly report heart beating or respiratory conditions, without enteral energy source. Implantable cardiac or respiratory sensors could ascertain lots of potential disease risk and give feedbacks and warnings timely. The implantable sensors had fidelity and accuracy in long-time monitoring. Meanwhile, the steadily working of them would not bring inconvenience to the movement and activities of human, which was superior to wearable biomedical monitoring systems. Without any external power supply, NGs would play great roles in the healthcare as self-powered implantable sensors in the future.

**Figure 9:**
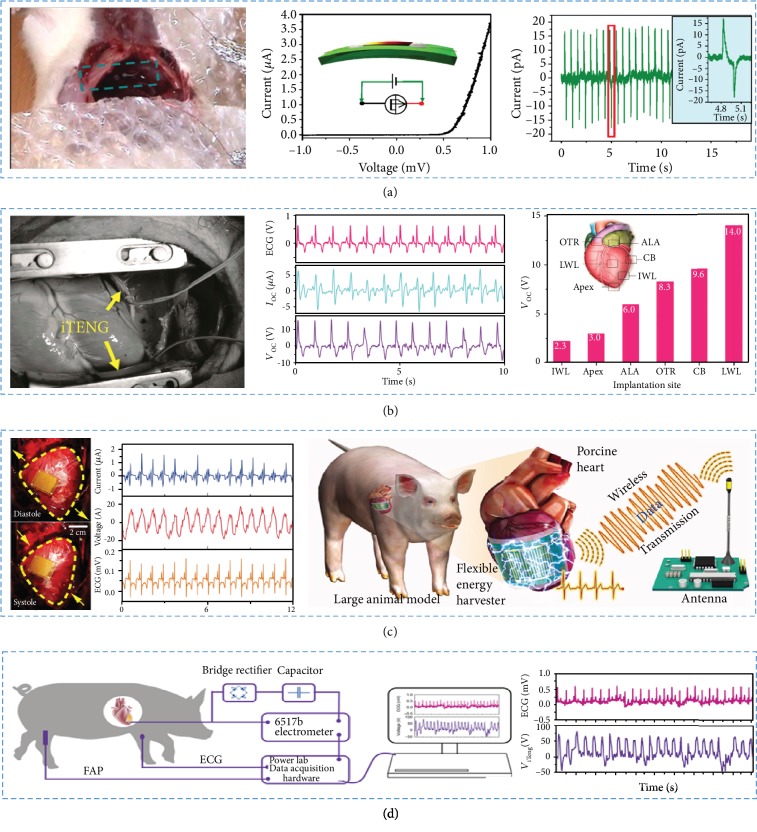
The devices were implanted in animals to measure the respiratory and heart beating. (a) A SWG attached to a live rat's diaphragm to sense the signal from the breath and heart beating. (b) iTENGs were implanted between the heart and the pericardium of swine to sense the ECG and breath. (c) The device was implanted *in vivo* to record ECG signals of the pig. (d) *In vivo* energy harvester was provided to measure the motion of the heart.

Basing on two-ends-bonded ZnO nanowires, Li et al. fabricated an implantable alternating current (AC) NG for the first time [70]. The fabricated AC generator has been set in the body and applied in biomechanical energy harvesting as shown in Figure 9(a). During the study, the implantable NG had been applied in scavenging energy from the breath and heartbeat of a live rat. The study provided a working strategy for harvesting the mechanical energy inside the body, containing cardiac motion, respiratory movement, and so on. In conclusion, the research played an important role in the development of implantable self-powered nanosystems.

In virtue of those animal experiments carried on the diaphragm, lung, and heart, Dagdeviren et al. demonstrated efficient energy conversion devices with the advanced materials and electronics [45]. The energy converter took advantage of the natural contractile and relaxation motions from organs which had the same size with human scales. Combining with microbatteries and rectifiers, the integrated flexible system could match well with the beating heart via medical sutures and operation in an efficiency of 2%. In summary, the devices provided sufficient power outputs for operating pacemakers, with or without the help of batteries.

The implantable TENG was fabricated by Zheng et al. and implanted between the heart and the pericardium [104]. As the heart contracted and relaxed periodically, the iTENG could contact and separate with the heart (Figure 9(b)). The output electrical signals were highly matched with heart rate, which could be applied in heartbeat monitoring. Furthermore, the respiratory movement could establish a close relationship with electrical-generating process. According to the result, the amplitude of electrical output was composed of the breath cycle. Therefore, the iTENG had a great potential in heart or respiratory monitoring.

Kim et al. prepared a PMN-PZT energy harvester with great performance to drive the wireless communication in the healthcare system as shown in Figure 9(a) [58]. The device was so biocompatible and flexible that it could be attached on the porcine heart to obtain the energy from heartbeats. Therefore, the devices implied great possibility as biomedical applications.

In the previous study, the researchers had evaluated that the self-powered implantable medical electronic devices could be applied in biomechanical energy harvesting from heart beating, breathing, and blood flowing. Based on this kind of implantable electronics, Ouyang et al. fabricated a fully implanted symbiotic pacemaker based on iTENGs [152]. The iTENGs could serve not only as an energy harvester but also as an energy storage along with cardiac pacing. As shown in Figure 9(d), the symbiotic pacemaker was implanted on a porcine and could successfully correct sinus arrhythmia and prevent deterioration. The implanted symbiotic pacemaker provided a new approach for endocardial pacing.

## 5. Conclusions and Prospects

Collecting body energy for powering implantable and wearable electronics has great practical significance, for there are large quantities of power sources wasting worthlessly all the time. A variety of tactics have been invented to harvest body energy, such as NG, automatic watch, and biobatteries. This review summarized the NGs acting as active wearable sensors such as touch sensors, motion sensors, strain sensors, weight sensors, temperature sensors, sound sensors, and implantable sensors for monitoring blood pressure, cardiac signal, and respiratory signal. Before that, we illuminated the principle and materials of NGs. Meanwhile, the fabrication strategy for sensors has also been presented. The NGs were fixed on or implanted into the body, converting the mechanical energy or thermal energy to electric signals.

NGs can serve as wearable and implantable sensors for detecting environmental stimulation, pressure fluctuation, and mechanical triggering without external power, which thus has immense potential for infrastructure monitoring, physiological characterization, security systems, and human-machine interfacing. However, the development of NGs for active sensors is still on the way; some problems are urgent to be solved. First, novel materials need to be developed which give the NGs properties of lightweight, flexibility [153], and good electrical performance so that they can preferably physically match with the human body or be implanted into certain parts of the body for a long time. Since biocompatibility is vitally important for implantable sensors or electronics, the implanted devices should be prepared with biocompatible materials. Second, since the liquid surroundings can screen the charges generated by the NG, soft encapsulation technique should be realized to maintain the function of NG. In addition, the structure of the NG should be optimized to decrease output attenuation after being packaged and implanted. Finally, the output of NG needs to correspond with the physiological signal. For wearable NGs, the materials, structure, encapsulation, and signal veracity are also very important.

NGs can act as distributed mobile/wearable sensors. A prospective research of NG-based implantable sensors should be developed for sensors of organs in the body such as the intestinal tract, stomach, spleen, liver, lung, and cranium and related physiological information and disease surveillance. Concentration and composition sensors such as tear, seat, and urine and light sensors for wearable application are also critical for life and health. In addition, the evaluation system of NG-based sensors such as flexibility, stability, sensitivity, weight, size, and practical potential should be established pressingly. Meanwhile, extraction and analysis methods of NG signals are needed since the sensors usually receive different sensing information at the same time. Finally, the NG can also act as the micropower source for wireless-distributed implantable/wearable sensors. The self-powered sensors will promote the sensor networks, IoT, and intelligent medical electronics. They also possess great merits in biomedical science, MEMS, robotics, and smart e-skin among other fields.

## Figures and Tables

**Table 1:** The characteristics of wearable and implantable PENGs.

	Type feature	Material	Size (cm)	Output voltage (*V*)	Function
PENG	Inorganic	PZT [45, 57] ZnO [56, 68, 70] PMN-PZT-Mn [45] Se [61] Te [62] BaTiO_3_ [59] MoS_2_ [60]	1 × 1.5 [45] 1.5 × 10^−5^ (*Φ*) [56] 2 × 3 [58] 7 ± 3 × 10^−7^ (*Φ*) [61] 5 × 10^−5^ (*Φ*) [62] 1 − 8 × 10^−5^ (*Φ*) [70]	0.0235 [56], 0.3-1.85 [57], 17.8 [58], 105 [59], 18 [60], 0.45 [61], 0.1 [68], 0.05 [70]	Power source [57, 58] Sensor [45, 56, 59–62, 68, 70]
	Organic	HS-FPCS [47] PVDF [49, 63, 64, 66, 71] Chitin [51] PTFE-FEP [68] P(VDF-TrFE) [67, 69]	0.5 × 0.5 [49] 1 × 1.5 [51] 1.7 (*Φ*) [71] 2.5 × 2.6 [63] 1 [66] 1 × 1 [67] 5 × 5 [68] 1.5 × 1.5 [69]	130 [47], 1 [51], 1.75 [63], -0.016-0.014 [66], 4.8 [67]	Power source [49, 63, 71] Sensor [47, 51, 64, 66–69]

**Table 2:** The characteristics of wearable and implantable TENGs.

	Type feature	Material	Size (cm)	Output voltage (*V*)	Function
TENG	Polymer-metal	PTFE-Al [73, 104, 105] FEP-Cu [74] Rubber-steel [75] Kapton-Cu [94, 106] PLGA-Au [96]	2 × 2 [73] 5 (*Φ*) [74] 1.2 × 1.2 [96] 3 × 2 [105] 7.5 × 5 [106]	116 [73], 17.5 [74], 200 [75], 1.52 [94], 28 [96], 45 [104], 10 [105]	Power source [74, 75, 96, 104] Sensor [73, 94, 105, 106]
	Polymer-polymer	PET-PTFE [83] Nylon-PDMS [84] PDMS-PVDF [87] FEP-latex [97] PDMS-FEP [98]	8 × 3 [83] 5 [87] 2 (*Φ*) [97] 3.3 × 3.3 [98]	300 [84], 14 [87], 14.5 [97], 14 [98]	Power source [84, 87] Sensor [83, 84, 97, 98]
	Polymer-semiconductor	PTFE-PVDF [85] PVDF-PA6 [99]	6 × 3 [85] 6 × 4 [101] 1.5 × 1.5 [107]	1000 [99], 0.95 [101]	Power source [99] Sensor [85, 99, 101, 107, 108]
	Others	PET-conductive fiber [88] Silicon rubber-skin [90] PDMS-graphene [91] Glass-silk [109]	200 × 150 [88] 3 × 3 [90] 2 × 1.8 [109]	3 [88], 95 [90], 15.1 [91]	Power source [90, 109] Sensor [88, 91, 103, 109]

**Table 3:** The characteristics and classification of Hybrid NGs.

	Type feature	Material	Size (cm)	Output voltage (*V*)	Function
Hybrid	TENG-PENG	PVDF [110] PVDF-n-PDMS [112] BCZT/PVDF-HFP [113] P(VDF-TrFE)-PU [116]	1.5 × 1 [111] 7 × 5 [112] 3 × 3 [113] 2.5 × 3 [116]	12 V [110], 18 V [111], 0.15 V [116]	Power source [110–113] Sensor [113, 116]
	TENG-PENG-EMG	Nylon-PTFE [114] PZT-PTFE [115]	5 × 3 [114] 1.4 (*Φ*) [115]	75 V [114]	Power source [114, 115]
	TENG-EMG	Kapton-PTFE [118] FEP-Cu [119]	6.7 × 4.5 [118] 65 (*Φ*) [119]	4 mA [118] 9.5 V and 150 V [119]	Power source [118, 119]
	PENG-PYENG-TENG	Ag-coated fabric fiber [117]	12 × 3 [117]	—	Sensor [117]

**Table 4:** The summarization for the development of wearable sensors based on NGs.

Type feature	Tactile sensors	Motion sensors	Strain sensors	Others
Size (cm)	1.5 × 1.5 [124] 1 × 1 [128] 2 × 1.5 [129] 6 × 3 [130] 5 × 5 [131]	7.1 (*Φ*) [119] 150 [133] 2 × 6 [134] 10 × 6 [135] 0.01 (thick) [136] 20.32 × 29.46 [137] 3 × 1.6 [138]	1 × 1 [106] 2 × 1.8 [109] 2 × 1 [139] 1 × 2.5 [141] 5 × 5 [143]	7.5 × 5 [106] 25 [145] 1.5 × 1.5 [107] 2.5 × 3 [116] 2 × 3 [147] 3.5 × 3.5 [148]

Sensitivity/output voltage	0.99 V·kPa^−1^ [128] 0.39-1.46 V·N^−1^ [129] Uniaxial strain 1160% [132]	0.06 V·N^−1^ [133] 45.5 mV·K^−1^ [134] Over 2000 V·m^−2^ [137] 72 V/*ε* (*ε* = Δ*L*/*L*_0_) [138]	2.0 V [106] 29.4 mV [139] 0.16 W·m^−2^ [143] 49.7 V [143] 1.2 V [144]	0.55 V·kPa^−1^ and 0.1 V·°C^−1^ [107] <0.1 K and >28.9 kPa^−1^ [147] 42 V [148]

Position/accessory	Finger [124–126, 128] Finger skin [127] Finger and hand [129] Hand [131, 132] Wrist [130]	Hand and chest [119] Sock [133] Elbow and wrist [134] Arm and leg [135] Wrist, foot, elbow, knee [137] Cap or jaw [138]	Joint [106] Forearm, shirt, pants [109] Abdomen [117] Thumb and wrist [140] Finger [141, 144] Cotton glove [142] Finger, elbow, arm, knee [143]	Elbow, leg, neck [106] Respirator [108, 145, 148] Finger [107] Waist and abdomen [116] Hand and fingertip [147]

Flexibility	Yes [124–132]	Yes [133–138] None [119]	Yes [109, 117, 140–144] None [106, 139]	Yes [108, 118, 147] None [107, 108, 145, 148]

Stretchability	Yes [124–126, 129–132] None [127, 128]	Yes [135, 138] None [119, 133, 134, 136, 137]	Yes [117, 140–144] None [106, 109, 139]	Yes [106, 147] None [107, 109, 110, 118, 148]

Types of nanogenerator	PENG [128, 132] TENG [125–127, 129–131] PYENG [124]	TENG [135, 136, 138] Hybrid [119, 133, 134, 137]	PENG [139, 144] TENG [106, 109, 140–143] Hybrid [117]	TENG [107–110] PYENG [147, 148] Hybrid [116]

**Table 5:** The summarization for the development of implantable sensors based on NGs.

Type feature	Blood pressure sensors	Cardiac and respiratory sensors
Size (cm)	1.7(*Φ*) [71] 3 × 2 [105] 1 × 1.5 [151]	2 × 3 [58] 1 − 8 × 10^−5^ (*Φ*) [70]

Sensitivity/output voltage	1.195 mV·mmHg^−1^ [151]	4.5 V [45] 17.8 V [58] 50 mV [70] 45 V [104] 187 V [152]

Position/accessory	Heart [71, 105] Heart and femoral artery [151]	Heart, lung, diaphragm [45] Heart [58, 152] Diaphragm [70] Between heart, pericardium [104]

Flexibility	Yes [71, 105, 151]	Yes [45, 58, 70, 104, 152]

Stretchability	None [71, 105, 151]	None [45, 57, 58, 70, 104, 152]

Types of nanogenerator	PENG [71] TENG [105, 151]	PENG [45, 58, 70, 104] TENG [152]
